# Dissections Illustrated

**Published:** 1893-06

**Authors:** 


*Dissections Illustrated.* By C. GORDON BRODIE, F.R.C.S., with Plates by PERCY HIGHLEY. In Four Parts. Part I.—The Upper Limb. London: WHITTAKER & CO. [1892.]

These plates, which are all prepared from original dissections, are very good and accurate. They are much like the well-known plates of Ford and Ellis, but not so large, being two-thirds only of the natural size; they are thus much more portable, and are well adapted for the use of students, who can make use of them as aids to dissection. We have not seen anything of the kind so well done, and can strongly recommend them to anyone wishing to refresh his memory on anatomical points without having recourse to a dissecting-room.

